# Cis-Acting Sequence Elements and Upstream Open Reading Frame in Mouse Utrophin-A 5'-UTR Repress Cap-Dependent Translation

**DOI:** 10.1371/journal.pone.0134809

**Published:** 2015-07-31

**Authors:** Trinath Ghosh, Utpal Basu

**Affiliations:** Department of Molecular Biology & Biotechnology, University of Kalyani, Kalyani, West Bengal, India; International Centre for Genetic Engineering and Biotechnology, ITALY

## Abstract

Utrophin, the autosomal homologue of dystrophin can functionally compensate for dystrophin deficiency. Utrophin upregulation could therefore be a therapeutic strategy in Duchenne Muscular Dystrophy (DMD) that arises from mutation in dystrophin gene. In contrast to its transcriptional regulation, mechanisms operating at post-transcriptional level of utrophin expression have not been well documented. Although utrophin-A 5'-UTR has been reported with internal ribosome entry site (IRES), its inhibitory effect on translation is also evident. In the present study we therefore aimed to compare relative contribution of cap-independent and cap-dependent translation with mouse utrophin-A 5'-UTR through m7G-capped and A-capped mRNA transfection based reporter assay. Our results demonstrate that cap-independent translation with utrophin-A 5'-UTR is not as strong as viral IRES. However, cap-independent mode has significant contribution as cap-dependent translation is severely repressed with utrophin-A 5'-UTR. We further identified two sequence elements and one upstream open reading frame in utrophin-A 5'-UTR responsible for repression. The repressor elements in utrophin-A 5'-UTR may be targeted for utrophin upregulation.

## Introduction

Duchenne Muscular Dystrophy (DMD) is the most frequent genetic disorder which is caused by mutation in X linked dystrophin gene [1, 2]. Dystrophin is the part of dystroglycan complex that links cytoskeleton with extracellular matrix. In absence of functional dystrophin, the sarcolemal integrity is lost and DMD arises. The chromosome 6 encoded utrophin structurally and functionally resembles dystrophin, although utrophin expression is reduced postnatally and confined preferentially at neuromuscular and myotendinous junctions in adult mice [3–7]. Overexpression of utrophin in dystrophin deficient mouse muscle fiber either through transgenic experiment or viral vector reduced dystrophic pathology [8–11]. Utrophin upregulation has therefore been considered as a strategy for functional substitution of defective dystrophin. For an effective therapy of DMD, utrophin expression however needs to be upregulated throughout the adult muscle fiber [10, 12–17]. Better understanding of molecular events regulating utrophin expression may lead to the development of strategies aimed at upregulation of utrophin in muscle fibers of DMD patients.

Expression of utrophin is driven by two different promoters, namely A and B although muscular expression is predominantly driven by promoter A [18, 19]. Although some of the regulatory mechanisms playing major roles during transcription via the utrophin-A promoter have been identified [20, 21], limited work focused on post-transcriptional and translational regulation of utrophin-A expression. AU-rich element in utrophin-A 3'-UTR negatively regulates mRNA stability [22]. Internal Ribosome Entry Site (IRES) has been reported to be associated with utrophin-A 5'-UTR and eEF1A2 is known to interact with it [23, 24]. Our previous work demonstrated that mouse utrophin-A mRNA is translationally repressed and this repression is mediated through its 5' and 3'-UTRs. Although the repression with 3'-UTR has been attributed to miRNAs and K-homology splicing regulator protein (KSRP) [25–27], lacuna exists in our understanding of exact inhibitory mechanism operated through 5'-UTR. The reported IRES activity associated with 5'-UTR and current skeptical views regarding cellular IRES [28] have made the scenario much more complicated.

The major criticism regarding cellular IRES is the approach used for their characterization. The DNA based dicistronic constructs usually used for IRES identification very often gives false positive results. The false positive results mostly associated with the presence of cryptic promoter and splice acceptor site in the sequence to be tested for IRES [28, 29]. Like most of the reported cellular IRESes, utrophin-A 5'-UTR was tested with the same approach. Miura et al ruled out the existence of cryptic promoter and splice variants with Northern blot, while first documented utrophin-A IRES [23]. However, given the sensitivity of Northern blot to detect transcript variant [28], it is rational to cross-check mouse utrophin-A 5'-UTR for IRES with advanced and rigorous strategy. Since A-capped mRNA is poorly translated through cap-dependent canonical mode, its expression provides reliable estimation of cap-independent translation. With A-capped mRNA transfection, in the present study we have demonstrated that utrophin-A 5'-UTR can be translated through cap-independent manner. Although cap-independent translation with utrophin-A 5'-UTR is relatively low compared to well-studied strong EMCV IRES, it plays significant role in the pretext of strong inhibition of cap-dependent translation. Focusing repression of cap-dependent translation, we have identified two cis-acting sequence elements in utrophin-A 5'-UTR. These elements along with one short open reading frame (ORF) in 5'-UTR repress cap-dependent translation.

## Materials and Methods

### Plasmid constructs

The full length mouse utrophin-A 5'-UTR was amplified with AATTCCATGGGTTGTGG AGTCGCCCTTCCC and AATTCCATGGCTTGAATGAGTTTCAGTATAATCCAAAG using cDNA prepared with total RNA obtained from mouse myoblast C2C12 cells and inserted into NcoI site of pGL3 control vector at upstream of firefly ORF. The EMCV IRES was amplified with primer EMCV_F: AAAACCATGGCCCTCTCCCTCCCCCCCCCCTAAC and primer EMCV_R: AAAACCATG GTGTGGCCATATTATCATCGTGTTTTTC using pIRES2-AcGFP1 vector (Clonetech) as template and cloned into NcoI site of pGL3 control vector (Promega). The EMCVmut is non functional IRES that contains a 61 nt deletion mutation within IRES [30, 31]. EMCVmut was made by amplifying upstream and downstream regions of the deleted sequence followed by annealing and elongation. Upstream region was amplified with EMCV_F and GGGTTAACCC CTTCTGGGCATCCTTCAGCCCCTTG. Primer pair EMCV_R and GGGTTAACCCCGAGGTTAAAAA AACGTCTAGGCCC were used for amplification of downstream sequence. Deletion of the following regions 125–255, 255–302, 303–352, 353–422 from 5'-UTR of utrophin-A mRNA were done with the similar strategy. Primers used for upstream and downstream amplification are given in S1 and S2 Tables. The deletion Δ423–507 was made by amplifying 1–422 nt using primer pair AATTCCATGGGTTGTGGAGTCGCCCTTCCC and AATTCCATGG TTCATGCTAGCCTGGACCATTTTTCA. Full length and deletion mutants of both utrophin-A 5'-UTR and EMCV IRES were inserted into NcoI site of pGL3 control vector. Point mutation at AUG425 in 5'-UTR was done by Phusion Site-Directed Mutagenesis Kit (Thermo Scientific) using oligos ggctagcaggtattcaAgctagcctggaccatttttc and GAAAAATGGTC CAGGCTAGCTTGAATACCTG CTAGCC.

### *In vitro* transcription

All constructs were amplified with a T7 containing forward primer and a T_50_ tailed reverse primer to prepare templates for *in vitro* transcription. *In vitro* transcription and m7G-capping reactions were carried out with T7 RNA Polymerase (NEB) and Vaccinia Capping enzyme (NEB) respectively according to the manufacturer’s protocol. ApppG (NEB) was added to transcription reaction mixture in a proportion of 10:1 to GTP for preparation of A-capped RNAs. m7G-cap and A-capped RNAs were purified with RNA purification kit (Macherey-Nagel).

### Cell culture and RNA transfection procedures

Mouse C2C12 myoblast cells (ATCC, Cat. No. CRL-1772) were cultured in DMEM supplemented with 10% FBS (Invitrogen). Exponentially growing cells were distributed in 24 well plate. 50,000 cells in 500 μl of antibiotic free media were plated in each well 24 hours before transfection. 0.5 ug of Fluc mRNA with full length utrophin-A 5'-UTR and equimolar amount of other Fluc mRNAs were transfected in each well with Lipofectamine 2000 (Invitrogen) according to manufacturer’s protocol. 1 ng/well Rluc mRNA was co-transfected with Fluc mRNAs. Opti-MEM I (Invitrogen) was used to dilute RNAs and Lipofectamine 2000.

In 24 well plate, either 20,000 cells or 50,000 cells were plated in each well 38 hours before transfection in order to study the effect of etoposide (Cipla). Lower cell density wells were used as control. For etoposide treatment the wells with higher cell density were used. In culture media etoposide (100 μM final concentration) was added 6 hours after platting. Cell densities become approximately the same before transfection in control and treated sets. Transfection was performed as described above.

### Luciferase assay

Two hours post transfection, cells were washed with PBS and then harvested. The luciferase activities were measured with Dual Luciferase Assay Kit (Promega) according to manufacturure’s protocol.

### qPCR for luciferase and β-actin

DNase I treated 2 μg of total RNA isolated from transfected C2C12 cells was reverse transcribed with oligo dT using the MMLV High Performance Reverse Transcriptase (Epicentre), according to the manufacturer’s instructions. Quantitative PCR was done using primer pairs AAAGTTGCGCGGAGGAGTT and CCCTTCTTGGCCTTTATGAGG for luciferase or CGTGCGTGACATCAAAGAGAAGC and CCCAAGAAGGAAGGCTGGAAAAG for β-actin with KAPA SYBR FAST qPCR Kits (Kapabiosystems). Melt curve analysis for each reaction was performed to confirm the absence of nonspecific amplification. Target copy number was quantified using standard curve constructed with pGL3 (Promega) and β-actin amplicon cloned in pGEM-T vector (Promega) for luciferase and β-actin respectively.

### Western blotting

Cells were lysed with NP-40 lysis buffer (50 mM Tris-HCl pH 8, 150 mM NaCl, 1% NP-40) containing 1 mM PMSF (Sigma) and centrifuged to remove debris. Protein concentration in supernatant was assayed using a Quick Start Bradford Protein Assay reagent (Bio-Rad). Each sample containing 90 μg of protein was resolved on 3–20% SDS-PAGE. Upon transfer, Western blot was done with mouse monoclonal anti-utrophin (MANCHO03 clone 8A4, developed by Glenn E. Morris and obtained from the Developmental Studies Hybridoma Bank at the University of Iowa), rabbit anti-eIF4G (sc-11373, Santa Cruz Biotechnology) and mouse anti-β-actin (sc-81178, Santa Cruz Biotechnology) antibodies.

## Results

### Relative contribution of cap-dependent and cap-independent translation with mouse utrophin-A 5'-UTR

**Fig 1:**
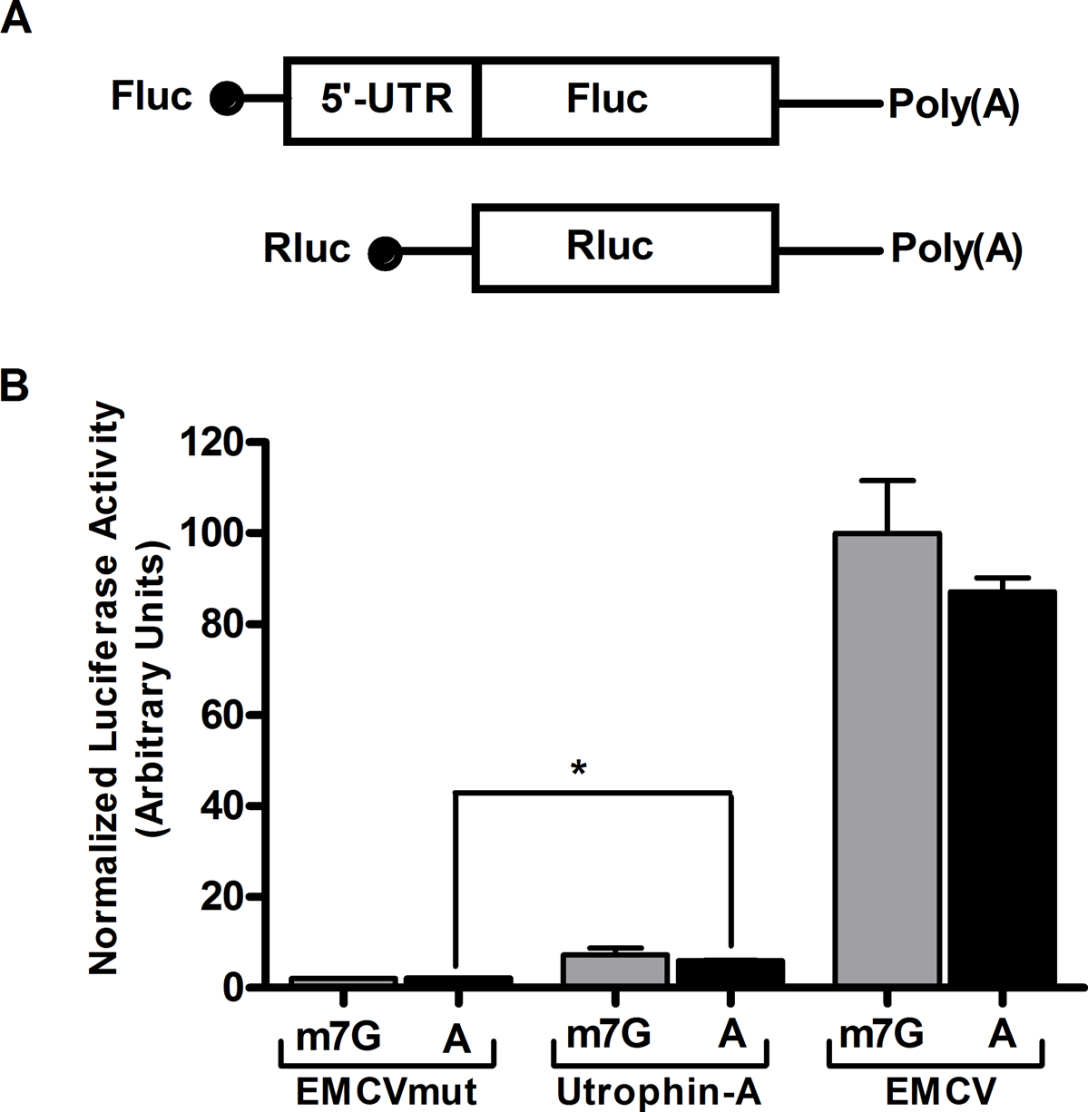
Comparison between cap-dependent and IRES dependent translation of utrophin-A 5'-UTR containing reporter. (A) Schematic presentation of monocistronic mRNA pairs used in the assay. Fluc: Firefly luciferase, Rluc: Renilla luciferase. To normalize activities of m7G-capped and A-capped Fluc constructs containing the tested 5'-UTRs, the m7G-capped Rluc was used as reference. (B) Comparison of indicated m7G-capped vs. A-capped monocistronic transcripts in the RNA transfection assay for mouse C2C12 cells, where utrophin-A 5'-UTR is compared with EMCV IRES. The relative A-capped Fluc/m7G-capped Rluc luciferase activity indicates IRES activity of the 5'-UTRs. Results presented as mean±SD (n = 6). Student’s *t* test was used to analyze the data. The asterisk denotes the statistically significant (P<0.0001) IRES activity of utrophin-A 5'-UTR compared to negative control EMCVmut.

Miura et al long ago reported IRES in utrophin-A 5'-UTR and upregulation of utrophin during muscle regeneration and glucocorticoid treatment has been attributed to activation of this IRES [23, 32]. However, recently many authors questioned the existence of IRES in cellular mRNAs primarily because of the apparent flaws in the experimental designing. Most of the cellular IRES elements have been reported based on the DNA based dicistronic constructs, where the sequence to be tested was inserted between two reporter cistrons. It has been assumed that the translation of first one is driven by cap, while the second one if translated at all would be stimulated by cap-independent recruitment of ribosomal machinery over the inserted sequence. The dicistronic DNA based transfecton in cultured cells are now facing various questions as they can produce artifacts in determining IRES activity of a certain sequence [28, 29]. More rigorous test based on RNA transfection has therefore been proposed to determine IRES activity of a particular sequence. Previous reports of IRES in 507 nt utrophin—A 5'-UTR were solely based on DNA based dicistronic constructs, although the authors demonstrated absence of alternate transcript and splice variant through Northern Blot [23]. We therefore questioned whether utrophin-A 5'-UTR can pass more rigorous RNA based IRES test. In C2C12 myoblast cells we transfected *in vitro* transcribed, A-capped and poly A tailed firefly luciferase mRNA with utrophin-A 5'-UTR at its upstream. m7G-capped renilla luciferase was used as transfection control. A-capped mRNA cannot be translated by cap-dependent manner and the reporter activity would only be detected if the mRNA is translated through cap-independent fashion. In parallel we used m7G-capped mRNA of luciferase ORF having upstream utrophin-A 5'-UTR. A-capped transcripts with EMCV IRES and EMCVmut inserted at the upstream of firefly luciferase reporter ORF were used as positive and negative control respectively. Two hour upon transfection of A-capped mRNA in C2C12 cells, we detected very high reporter activity with EMCV IRES in contrast of EMCVmut (Fig 1). For comparative study we transfected equimolar amount of m7G-capped mRNAs as well. For EMCV IRES, the A-capped reporter mRNA showed ~90% activity compared to m7G-capped mRNA. The reporter activity of EMCVmut was found to be very low both with m7G and A-capped mRNA as expected. Regarding mouse utrophin-A IRES, we detected about 7% activity compared to EMCV IRES with A-capped transcript. Unlike viral IRESes, the cellular IRESes are weak and reported cellular IRESes showed similar level of activities in previous study [33]. Moreover, with A-capped transcript, we detected almost 80% reporter activity compared to m7G-capped transcript having utrophin-A 5'-UTR (Fig 1). It therefore clearly demonstrates that ORF downstream of 5'-UTR is translated mostly via cap-independent manner. The overall low activity of reporter also indicates extreme repression of cap-dependent translation with utrophin-A 5'-UTR.

**Fig 2:**
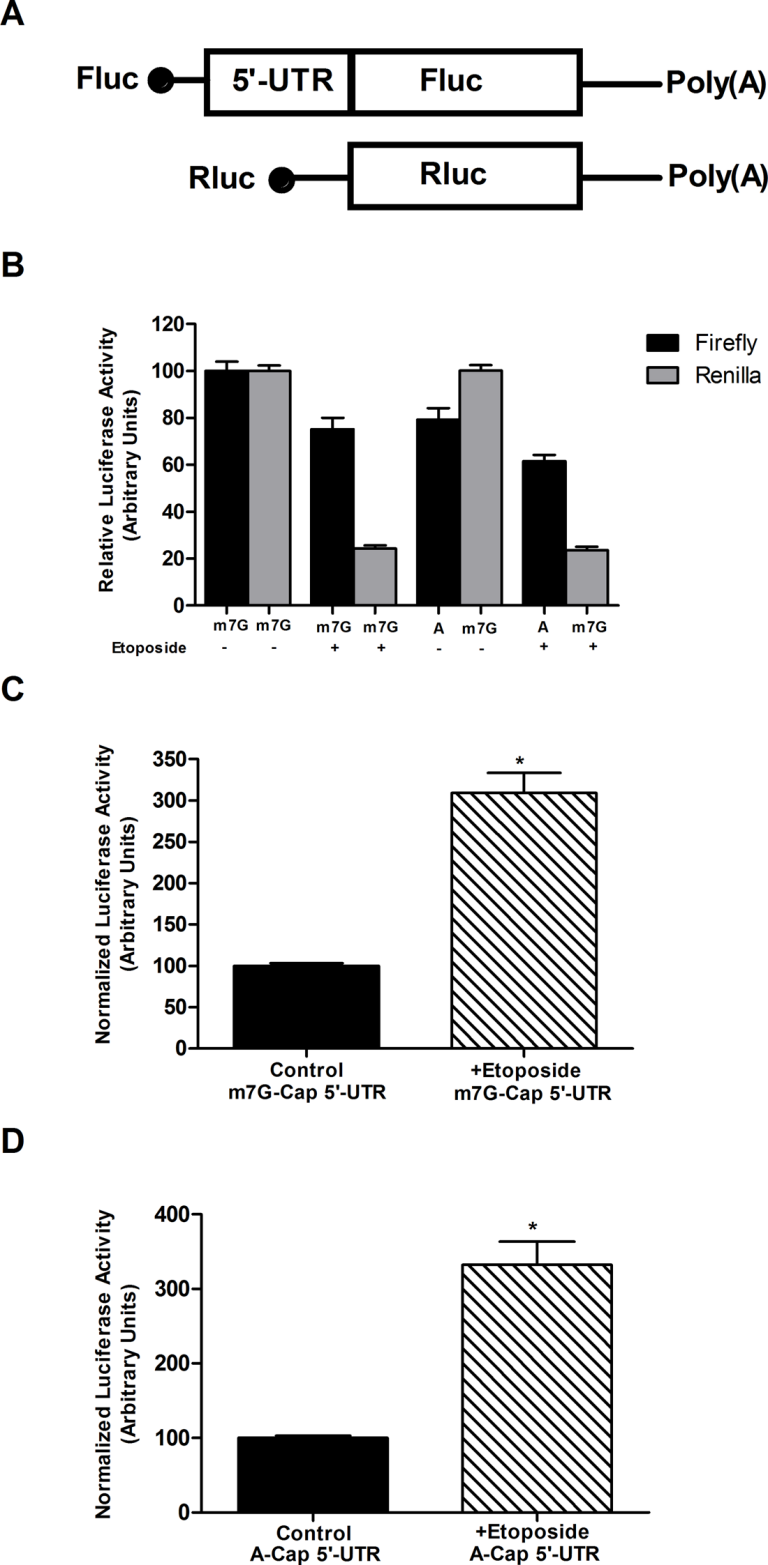
Translation of utrophin-A 5'-UTR with reporter in presence of etoposide. (A) Schematic presentation of monocistronic mRNAs used in the assay. Fluc: Firefly luciferase, Rluc: Renilla luciferase. (B) m7G-capped renilla and either m7G or A-capped firefly with utrophin-A 5'-UTR were co-transfected in etoposide treated and control cells. The individual activities of Fluc and Rluc in each set were presented as mean±SD (n = 6). The activities of renilla and firefly from m7G-capped transcripts in absence of etoposide were set to 100%. To normalize activities of Fluc constructs containing the utrophin-A 5'-UTRs, the m7G-capped Rluc was used as reference. The results presented in B were used to construct graph in C and D. (C) The normalized Fluc activity driven by m7G-capped Fluc with utrophin-A 5'-UTR transcripts in presence and absence of etoposide as represented in B. Normalized Fluc activity of m7G-capped utrophin-A 5'-UTR containing reporter was increased as a result of inhibition of m7G-Rluc expression indicating its cap-independence. (D) The normalized firefly activity driven by A-capped Fluc with utrophin-A 5'-UTR transcripts in presence and absence of etoposide as shown in B. Etoposide treatment similarly augmented the normalized Fluc activity of A-capped reporter with utrophin-A 5'-UTR. Student’s *t* test was used to analyze the data presented in (C) and (D). The asterisks denote the statistically significant (P<0.0001) difference in normalized luciferase activity (n = 6).

We wanted to cross check cap-independent translation of reporter having utrophin-A 5'-UTR with etoposide treated C2C12 cells. Etoposide treatment inhibits cap-dependent translation by inducing partial cleavage of eIF4G and sequestering eIF4E, the factors essential for cap-dependent translation initiation. We transfected A and m7G-capped mRNA in etoposide treated C2C12 cells to study whether utrophin-A 5'-UTR can sustain expression while cap-dependent translation is compromised. m7G-capped renilla reporter transcript was co-transfected to normalize firefly activity for all experimental sets (Fig 2) [34]. In all etoposide treated sets, m7G-capped renilla expression was severely reduced as its translation is solely driven by cap. In contrast, reduction in expression of luciferase driven by m7G-capped firefly with utrophin-A 5'-UTR was much less indicating its cap-independent translation. When A-capped firefly with utrophin-A 5'-UTR was used, etoposide treatment reduced the luciferase activity to some extent, but not as much as m7G-capped renilla transcript (Fig 2B). Hence, normalization against the activity driven by m7G-capped renilla, firefly with both A and m7G-capped utrophin-A 5'-UTR showed increased activity in etoposide treated cells compared to control (Fig 2C and 2D). We have also confirmed that etoposide treatment reduces eIF4G in C2C12 cells as evident from Western blot. In the same experimental set, we noticed decreased expression of β-actin, whereas utrophin expression remained almost unaltered after etoposide treatment for 38 hours (S1 Fig).

Our previous work demonstrated overall inhibitory role of utrophin-A 5'-UTR in translation [25]. However inhibition even in presence of IRES or cap-independent translation, remained unexplained. Our present result demonstrates that utrophin-A 5'-UTR although supports cap-independent translation, it is fairly weak. However it has important contribution, because of severe inhibition of cap-dependent translation.

### Sequence 1–125 and 255–302 inhibits m7G-cap-dependent translation

**Fig 3:**
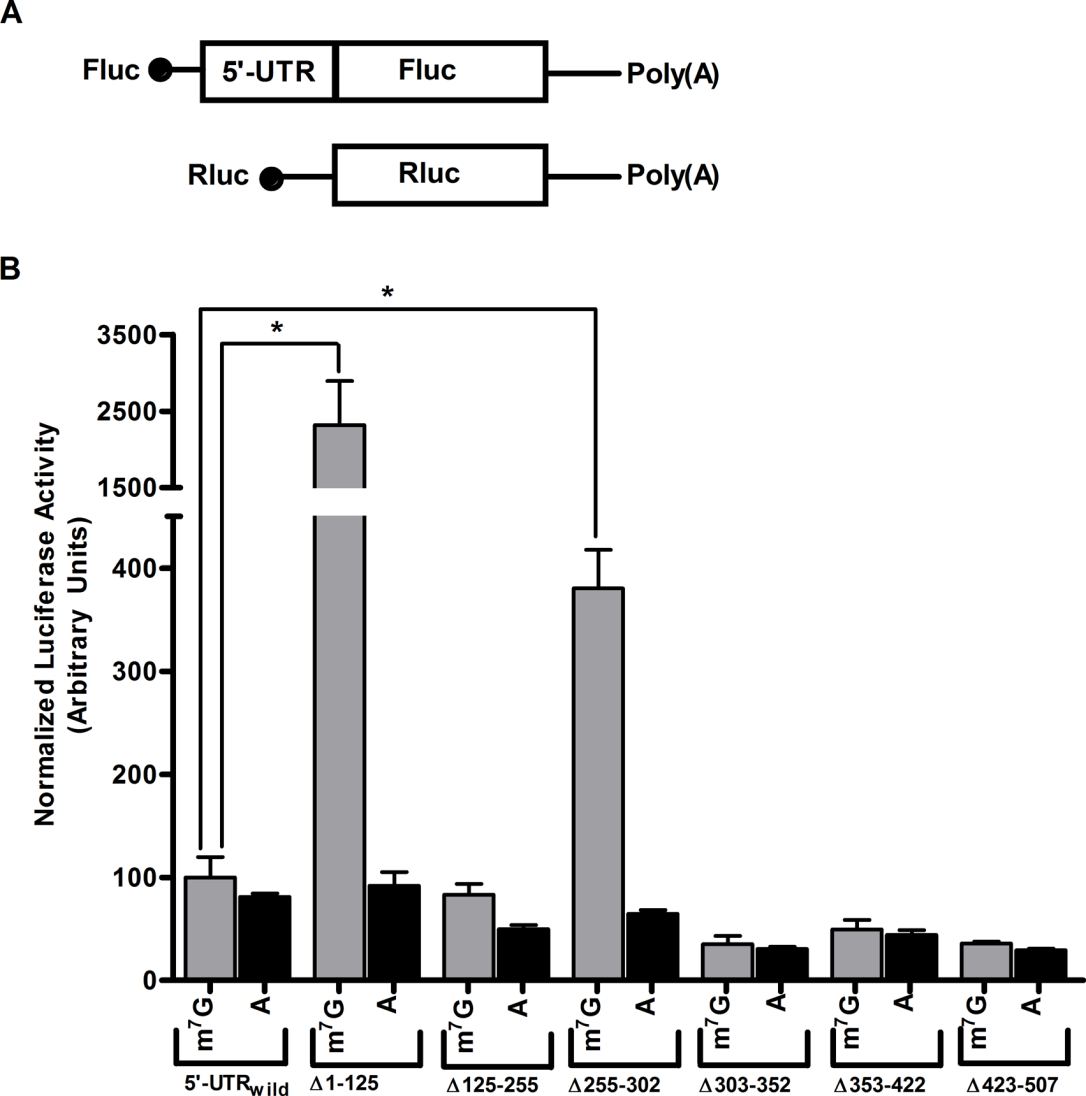
Deletion analysis of utrophin-A 5'-UTR for identification of repressor elements. (A) Schematic presentation of monocistronic mRNA pairs used in the assay. Fluc: Firefly luciferase, Rluc: Renilla luciferase. To normalize activities of m7G-capped and A-capped Fluc constructs containing the tested 5'-UTRs, the m7G-capped Rluc was used as reference. (B) Equimolar amount of m7G-capped and A-capped monocistronic transcripts were used in the RNA transfection assay with mouse C2C12 cells. Wild and Δ denote full length and deletion respectively. Results presented as mean±SD (n = 6). Student’s *t* test was used to analyze the data. The asterisks denote the statistically significant (P<0.0001) difference in relative luciferase activity of m7G-capped transcripts. Deletion of 1–125 nt and 255–302 nt upregulated m7G-cap-dependent expression. No deletion upregulated expression of A-capped transcripts.

In order to understand the role, if any, of different sequence elements in utrophin-A 5'-UTR mediated inhibition we first analyzed the utrophin-A 5'-UTR through Mfold, a web based programme (http://mfold.rna.albany.edu/?q=mfold) [35]. Using the Mfold predicted secondary structure (S2 Fig) for utrophin-A 5'-UTR with highest negative ΔG (-181.70 Kcal/mol) we have divided the 5'-UTR (1–507 nt) into six regions containing one or more stem loop structures. To identify secondary structural elements with regulatory role on translation initiation, we deleted different regions from 5'-UTR and deletion mutants were inserted upstream of firefly luciferase ORF. *In vitro* transcribed m7G-capped and A-capped mRNA corresponding to these deletion mutants were used for transfection in mouse myoblast C2C12 cells. m7G-capped transcript having deletion of 1–125 nt resulted ~25 fold increased reporter activity compared to full length utrophin-A 5'-UTR (Fig 3). The same transcript, when A-capped showed reporter activity almost indistinguishable from that of full length. Therefore 1–125 nt is inhibitory to cap-dependent translation and it has no function in IRES.

We have also detected a 48 nt sequence element present between 255 and 302 nt that inhibits m7G-cap-dependent translation. Deletion of this element resulted ~4 fold upregulation, when m7G-capped. With A-cap, the same deletion reduced the reporter activity to some extent showing its importance in IRES. However, this region is not as crucial as the first element in terms of its inhibitory effect on cap-dependent translation.

Having found differential expression of reporter from different deletion mutants as presented in Fig 3, we wanted to eliminate the possibility of differential RNA stability behind this observation. We therefore transfected equimolar amount of different *in vitro* transcribed deletion mutants in cultured C2C12 cells and total RNA was isolated 2 hours post transfection. No significant difference in abundance of different deletion mutants in cDNA obtained from total RNA was determined with real time PCR based quantification (S3 Fig).

### An upstream ORF in utrophin-A 5'-UTR represses translation

Existence of upstream ORF (uORF) has been reported to downregulate translation of protein-coding ORF. It has been found that 49% of human and 44% of mouse transcripts have upstream ORF [36]. Analysis of mouse utrophin-A 5'-UTR revealed an AUG at 425 nt. In the same frame there is UAG at 452 nt. Although the Kozak context of AUG_425_ is not ideal, we investigated whether this short open reading frame (ORF) exerts any regulatory effect in expression of downstream reporter ORF. We therefore mutated AUG_425_ into AAG.

**Fig 4:**
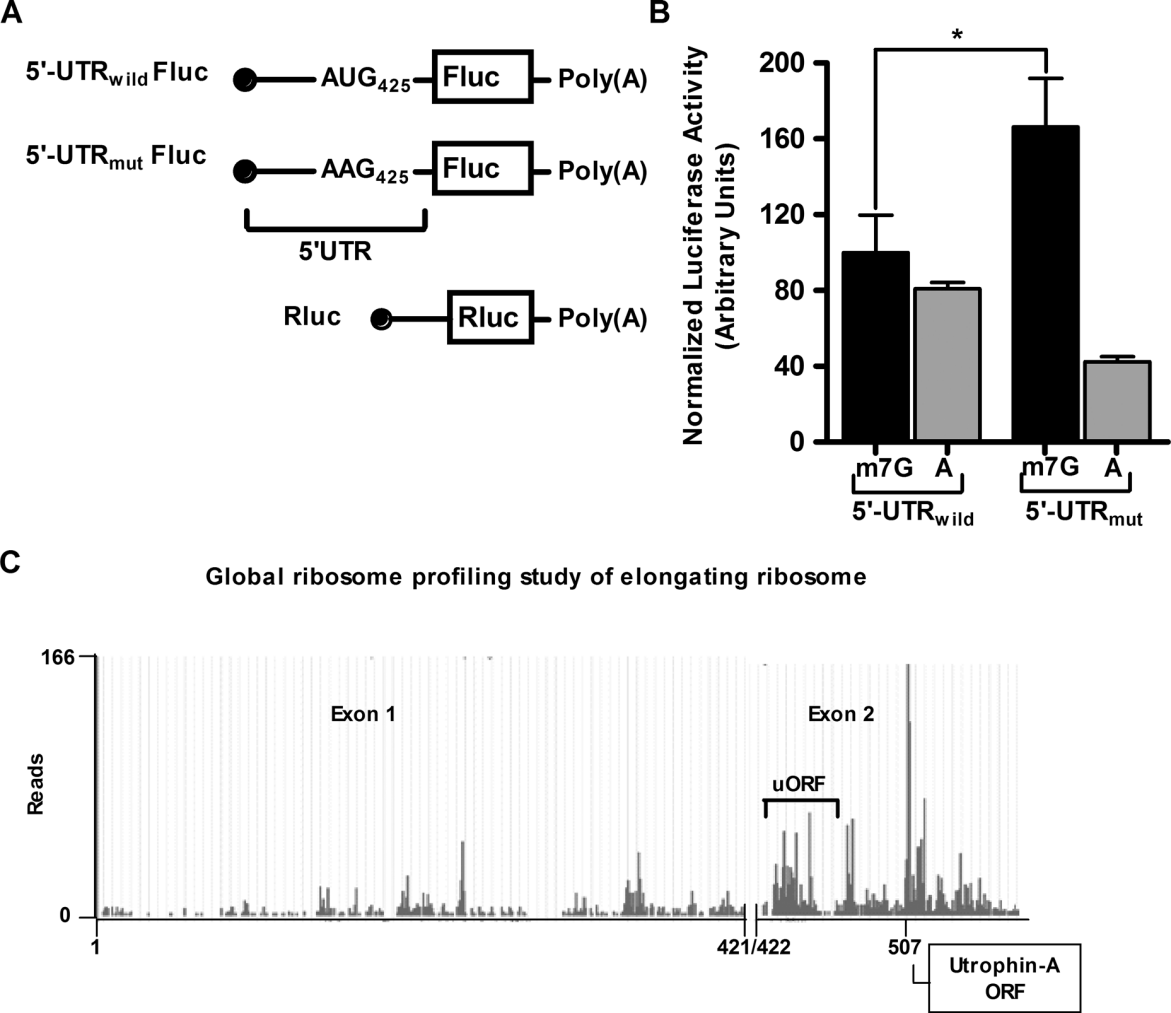
Role of upstream open reading frame on the utrophin-A 5'-UTR in m7G-cap-dependent translation. (A) Schematic presentation of monocistronic mRNAs used in the assay. Fluc: Firefly luciferase, Rluc: Renilla luciferase. To normalize activities of m7G and A-capped Fluc constructs containing the tested 5'-UTRs, the m7G-capped Rluc was used as reference. (B) Expression of m7G and A-capped monocistronic reporter transcripts with 5'-UTRwild and 5'-UTRmut were compared in the RNA transfection assay with mouse C2C12 cells. Upstream AUG in full length 5'-UTR (5'-UTRwild) was mutated to AAG425 in 5'-UTRmut. Results presented as mean±SD (n = 6). Student’s *t* test was used to analyze the data. The asterisk denotes the statistically significant (P<0.001) difference in normalized luciferase activity. (C) Results of global ribosome profiling study of elongating ribosome available at GWIPS-viz (http://gwips.ucc.ie) showed sufficient ribosome accumulation at upstream open reading frame (region 425–452 nt on utrophin-A 5'-UTR) as indicated by higher reads at this region.

Expression of *in vitro* transcribed m7G as well as A-capped, poly A tailed reporter RNA with firefly luciferase ORF having full length utrophin-A 5'-UTR with AUG_425_ and AAG_425_ was studied in C2C12 cells. When m7G-capped, mRNA with AAG_425_ showed more than 1.6 fold activity compared to the mRNA with AUG_425_ two hours post-transfection in C2C12 cells. It is to be noted that with A-capped transcript having AAG425 no upregulation was observed (Fig 4). This result therefore demonstrates the inhibitory role of upstream ORF in cap-dependent translation with utrophin-A 5'-UTR.

Upstream ORF, if translationally active would recruit ribosome. In ribosomal profiling experiment, isolation of ribosome-protected RNA fragments followed by their sequencing provides genome wide ribosome occupancy [37]. Data of such profiling experiments are available in GWIPS-viz database (http://gwips.ucc.ie) [38]. We checked ribosome occupancy in utrophin-A 5'-UTR in genome browser. At AUG_425_, ribosome occupancy is ~25% compared to annotated utropin-A start codon (Fig 4). Therefore, uORF in utrophin-A 5'-UTR is capable enough to capture scanning ribosomes and contributes in inhibition of cap-dependent translation.

## Discussion

Understanding of molecular mechanisms regulating utrophin expression may have therapeutic benefit because of utrophin’s potential to functionally compensate dystrophin deficiency in DMD. Two to four fold upregulation of utrophin is believed to be sufficient for complete amelioration of dystrophic phenotype in the disease [8, 39]. Although transcriptional control of utrophin expression is relatively well documented, its upregulation level sufficient for therapeutic benefit has never been reached. This may be due to inefficient translation of utrophin-A, the muscle specific isoform. Translational repression of utrophin-A is mediated through two major components: 3' and 5'-UTRs. Regulation through 3'-UTR could at least partially be explained by miRNA targets and AU rich element [22, 25–27]. However, 5'-UTR mediated repression of utrophin-A is quite puzzling, primarily because of its reported IRES activity [23]. In the same 5'-UTR simultaneous existence of inhibitory property and IRES, the *cis*-acting element mostly associated with translational upregulation demands in depth understanding. The present work is an effort towards this direction.

The evidence in favor of utrophin-A IRES came from DNA based dicistronic construct based assay. In the dicistronic construct insert to be tested is flanked between two reporter ORFs and two cistrons remain under the control of single but strong promoter. Expression of second cistron has been considered as the proof of IRES activity. In this strategy although second cistron is expressed for IRES elements, cryptic promoter and alternative splicing may also lead to the same. Therefore all the IRESes identified through dicistronic DNA construct based strategy, especially of cellular origin are now under question. We therefore tested utrophin-A 5'-UTR with more stringent strategy [28, 29, 33]. Luciferase reporter having 5'-UTR was *in vitro* transcribed, either m7G-capped or A-capped and then expression of reporter was monitored upon transfection in cultured cells. A-capped RNA cannot be translated through cap-dependent mechanism and therefore any activity of reporter represents its cap-independent translation. With A-cap we detected almost 80% activity of m7G-capped transcript (Fig 1). However the overall activity is quite low compared to EMCV IRES. We therefore conclude that firstly, utrophin-A 5'-UTR although confers IRES activity, it is not as strong as EMCV counterpart and secondly, its contribution becomes enormous because of severe repression of cap-dependent translation.

In order to investigate utrophin-A 5'-UTR mediated inhibition of cap-dependent translation, we asked whether any region within it confers repressor activity. With deletion mutagenesis, we addressed this issue using *in vitro* transcribed reporter RNA. Transfection based reporter assay identified two regions whose deletion upregulated expression of downstream reporter with m7G-capped transcript. These two sequences in utrophin-A 5'-UTR therefore inhibit cap-dependent translation. Our result suggests that ~125 nt long region at 5’terminus is crucial for severe inhibition in cap-dependent and has no effect on IRES. Its deletion upregulated ~25 fold reporter activity. This 125 nt sequence has predicted complex secondary structure and presence of structural elements close to 5' end inhibits binding of 40S ribosomal subunit with cap [40]. Another region, present between 255–302 nt in 5'-UTR moderately inhibits cap-dependent translation, however it seems important for IRES (Fig 3).

Investigations on upstream ORF (uORF) within 5'-UTR for many transcripts have demonstrated their inhibitory effect on translation [41]. In mouse utrophin-A 5'-UTR a short uORF is present. We therefore asked whether this uORF in utrophin-A 5'-UTR confers any inhibitory effect on downstream ORF. Elimination of utrophin-A uORF upregulated downstream reporter activity with m7G-capped RNA. It therefore identified another inhibitory element in utrophin-A 5'-UTR (Fig 4).

In conclusion, the present study demonstrates that utrophin-A 5'-UTR supports cap-independent translation. Utrophin-A IRES although not as strong as EMCV IRES, in the context of severe repression of cap-dependent translation, plays crucial role. The inhibition of cap-dependent translation is mediated through two cis-acting sequence elements and one short uORF. Among these three elements the sequence element present at 5' terminus appears most potent. The observations presented in this paper may therefore lead to the development of strategies to de-repress utrophin translation.

## Supporting Information

S1 Fig Western blot analysis of utrophin, eIF4G and β-actin upon etoposide treatment in C2C12 cells. C2C12 cells were treated with etoposide at 100 and 200 μM concentrations for 38 hours. Expression of eIF4G was severely reduced upon etoposide treatment. Although the expression of β-actin was decreased, utrophin expression remained almost unaltered.
(TIF)

S2 Fig Mfold predicted secondary structure of utrophin-A 5'-UTR with highest negative ΔG (-181.70Kcal/mol). (TIF)

S3 Fig Stability of *in vitro* transcribed m7G-capped firefly luciferase ORF with upstream utrophin-A 5'-UTR and its deletion mutants in cultured C2C12 cells. Equimolar amount of i*n vitro* transcribed m7G-capped mRNAs were transfected in C2C12 cells and copy numbers of luciferase ORF (FLuc) and β-actin were quantified in cDNA obtained from total RNA isolated 2 hours post transfection. Luciferase copy number was normalized against β-actin copy number. Result presented as mean±SD (n = 3). Normalized copy number of luciferase from full length 5'-UTR containing reporter was set to 100%. One way ANOVA was used to analyze the data. No significant difference was found among deletion mutants.
(TIF)

S1 Table Combination of PCR primers for internal deletion in utrophin-A 5'-UTR. (DOCX)

S2 Table List of PCR primers for preparation of utrophin-A 5'-UTR deletion mutants. (DOCX)
