# Supplementary figures and images for: Cis-Acting Sequence Elements and Upstream Open Reading Frame in Mouse Utrophin-A 5'-UTR Repress Cap-Dependent Translation
Source: PLoS One. 2015 Jul 31;10(7):e0134809. doi: 10.1371/journal.pone.0134809 (PMC4521823; doi:10.1371/journal.pone.0134809)

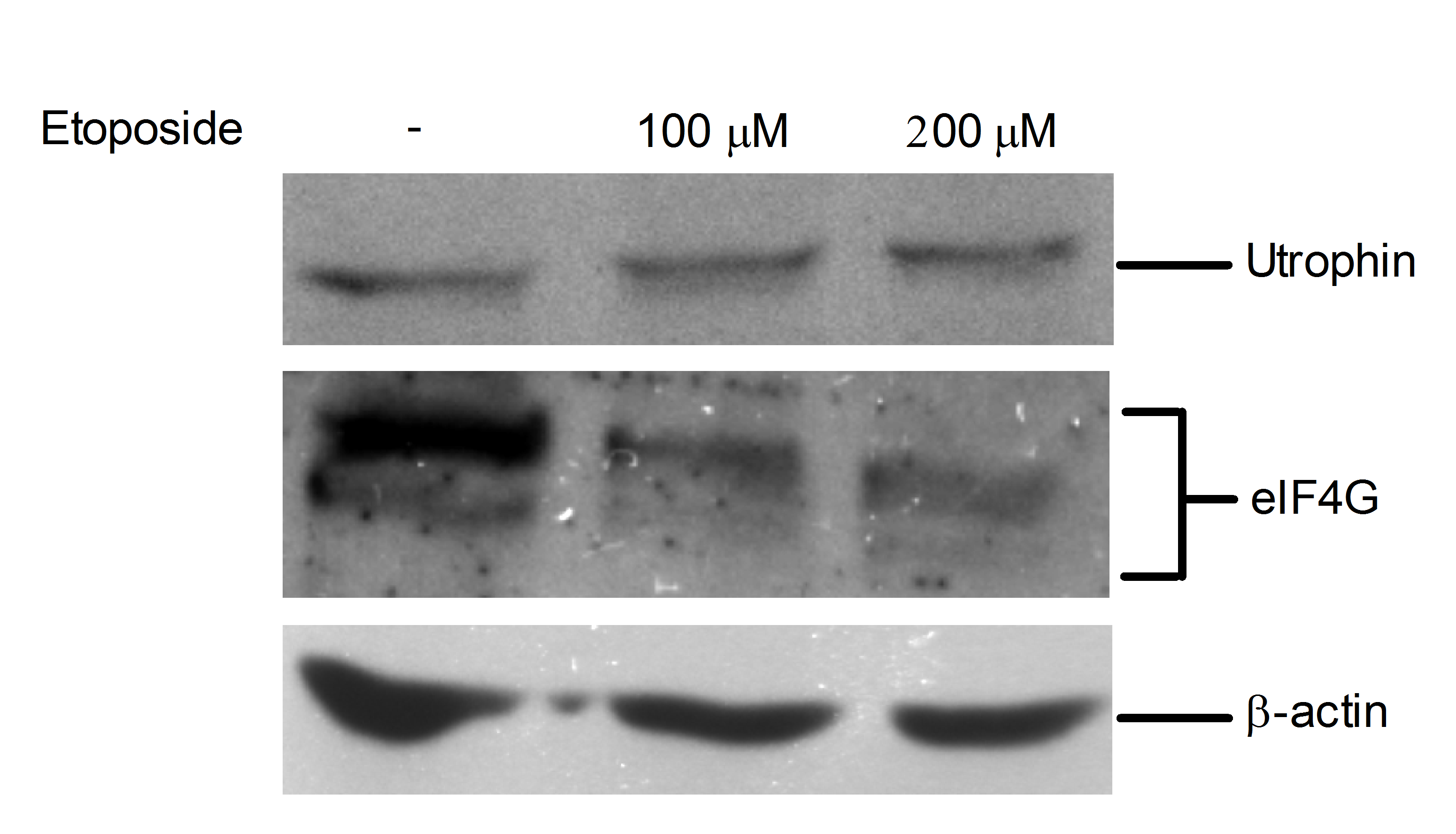

Supplement: S1 Fig — C2C12 cells were treated with etoposide at 100 and 200 μM concentrations for 38 hours. Expression of eIF4G was severely reduced upon etoposide treatment. Although the expression of β-actin was decreased, utrophin expression remained almost unaltered. (TIF) [file pone.0134809.s001.tif]

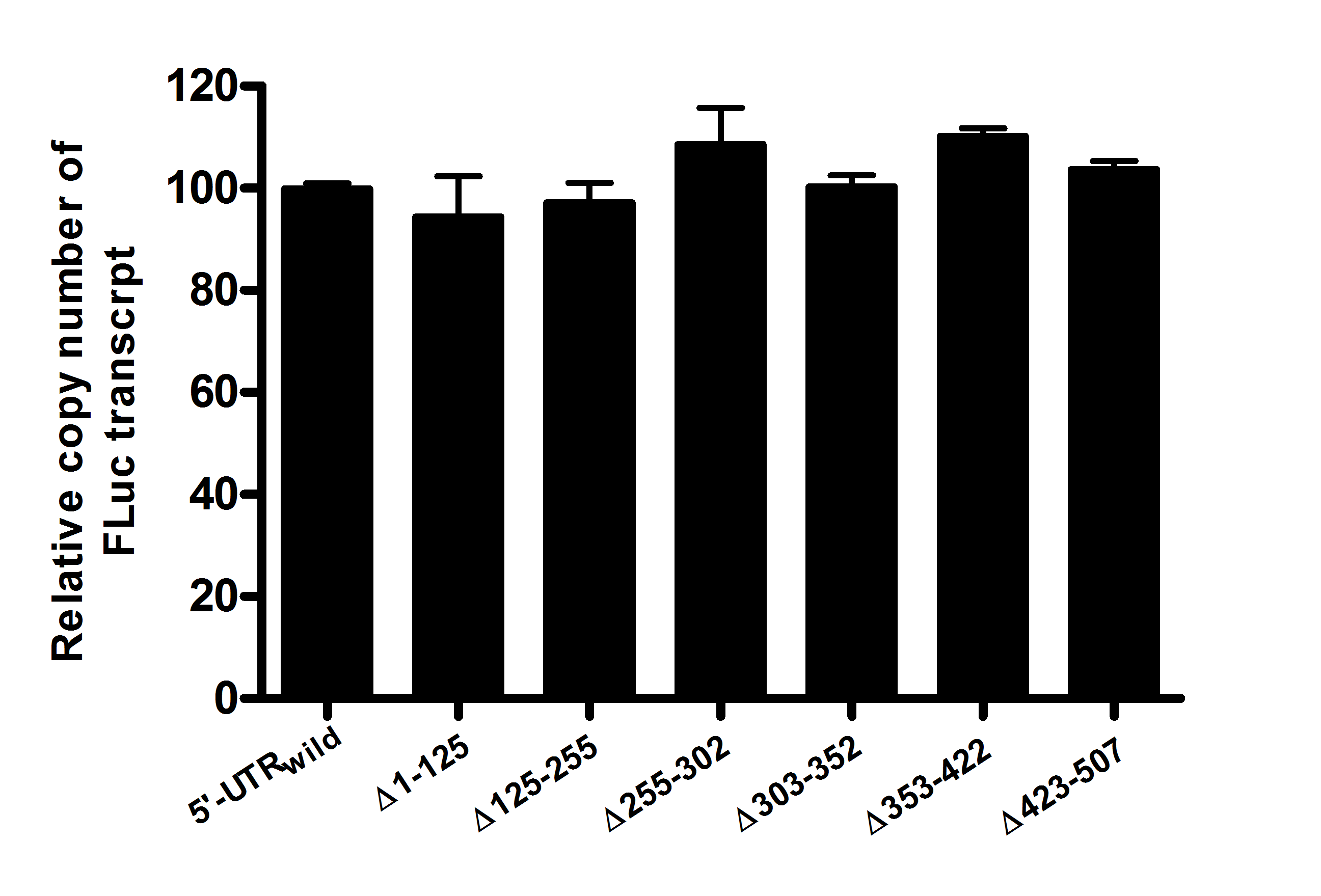

Supplement: S3 Fig — Equimolar amount of in vitro transcribed m7G-capped mRNAs were transfected in C2C12 cells and copy numbers of luciferase ORF (FLuc) and β-actin were quantified in cDNA obtained from total RNA isolated 2 hours post transfection. Luciferase copy number was normalized against β-actin copy number. Result presented as mean±SD (n = 3). Normalized copy number of luciferase from full length 5'-UTR containing reporter was set to 100%. One way ANOVA was used to analyze the data. No significant difference was found among deletion mutants. (TIF) [file pone.0134809.s003.tif]
