# Supplementary material for: Cis-Acting Sequence Elements and Upstream Open Reading Frame in Mouse Utrophin-A 5'-UTR Repress Cap-Dependent Translation
Source: PLoS One. 2015 Jul 31;10(7):e0134809. doi: 10.1371/journal.pone.0134809 (PMC4521823; doi:10.1371/journal.pone.0134809)
Supplement: S1 Table — (DOCX) [file pone.0134809.s004.docx]

**S1 Table. Combination of PCR primers for internal deletion in utrophin-A 5'-UTR.**

| Deleted region | Primers for upstream amplification | Primers for downstream amplification |
| --- | --- | --- |
| 125-255 | ∆125-255_R and Utrn_F | ∆125-255_F and Utrn_R |
| 255-302 | ∆255-302_R and Utrn_F | ∆255-302_F and Utrn_R |
| 303-352 | ∆303-352_R and Utrn_F | ∆303-352_F and Utrn_R |
| 353-422 | ∆353-422_R and Utrn_F | ∆353-422_F and Utrn_R |

Upstream and downstream PCR amplified regions were mixed, re-annealed and amplified with Utrn_F and Utrn_R primer pairs.
