# Supplementary material for: Cis-Acting Sequence Elements and Upstream Open Reading Frame in Mouse Utrophin-A 5'-UTR Repress Cap-Dependent Translation
Source: PLoS One. 2015 Jul 31;10(7):e0134809. doi: 10.1371/journal.pone.0134809 (PMC4521823; doi:10.1371/journal.pone.0134809)
Supplement: S2 Table — (DOCX) [file pone.0134809.s005.docx]

**S2 Table. List of PCR primers for preparation of utrophin-A 5'-UTR deletion mutants.**

| Name of primer | Sequence (5'-3') | Orientation  Forward(F)/ Reverse (R) |
| --- | --- | --- |
| Utrn_F | AATTCCATGGGTTGTGGAGTCGCCCTTCCC | F |
| Utrn_R | aattccatggcttgaatgagtttcagtataatccaaag | R |
| ∆125-255_R | GACACCCGATTGCGCCGCTCCCCGAGGTG | R |
| ∆255-302_R | GAAAGCCCGACAAGAGAAACGCCCCCTGACT | R |
| ∆303-352_R | TGGGGAGCTTGCCTCCAAAGGGGGGTGGGG | R |
| ∆353-422_R | CCAGGCTAGCATGATGGACGTTACAAGAATTTTTTT | R |
| ∆125-255_F | gagcggcgcaatcgggtgtcaattttg | F |
| ∆255-302_F | GGGGCCGTTTCTCTTGTCGGGCTTTCCACG | F |
| ∆303-352_F | ACCCCCCTTTGGAGGCAAGCTCCCCACCAC | F |
| ∆353-422_F | TTCTTGTAACGTCCATCATGCTAGCCTGGACCAT | F |

The Utrn_F and Utrn_R primers were used to amplify utrophin-A 5'-UTR (507 nt).
